# Participation in everyday activities and quality of life in pre-teenage children living with cerebral palsy in South West Ireland

**DOI:** 10.1186/1471-2431-8-50

**Published:** 2008-10-31

**Authors:** Vicki Mc Manus, Paul Corcoran, Ivan J Perry

**Affiliations:** 1 School of Nursing and Midwifery, UCC, Cork, Republic of Ireland; 2 School of Public Health, UCC, Cork, Republic of Ireland; 3 National Suicide Research Foundation, Cork, Republic of Ireland; 4 School of Public Health, UCC, Cork, Republic of Ireland

## Abstract

**Background:**

Cerebral palsy (CP) is the most common cause of physical disability in children but its impact on quality of life is not well understood. This study examined participation in everyday activities among children without CP and children with mild, moderate and severe impairment due to CP. We then examined ten domains of quality of life in children with CP and investigated whether participation in everyday activities was associated with improved quality of life independent of gender, age and level of impairment.

**Methods:**

This was a cross-sectional study of children aged 8–12 years based on two questionnaires, frequency of participation (FPQ) and KIDSCREEN, completed by parents of 98 children on the South of Ireland Cerebral Palsy Register (response rate = 82%) and parents of 448 children attending two Cork city schools (response rate = 69%) who completed one questionnaire (FPQ). Multiple linear regression was used: firstly to estimate the effect of severity of CP on participation in everyday activities independent of age and gender and secondly we estimated the effect of participation on quality of life independent of age gender and level of impairment.

**Results:**

Participation in 11 of the 14 everyday activities examined varied across the children without CP and the children with varying severity of CP. In general, increased impairment decreased participation. Independent of age and gender, there was a highly significant decrease in overall participation with a fall of -6.0 (95% CI = -6.9 to -5.2) with each increasing level of impairment. The children with CP generally had high quality of life. Increased impairment was associated with diminished quality of life in just two domains – Physical well-being and Social support and peers. Overall participation in everyday activities was significantly associated with quality of life in 3 of the 10 domains (Physical well-being, Social support and peers & Moods and emotions) in analysis adjusted for gender age and level of impairment.

**Conclusion:**

While increased impairment due to CP restricts participation in the majority of everyday activities, the level of participation has a limited effect on the quality of life of the children with CP in age 8–12 years.

## Background

Cerebral palsy (CP) is a chronic condition occurring in 2 to 3 per 1000 live births across Europe [1]. Defined as *'a permanent disorder of the development of movement and posture, causing activity limitations that are attributed to non-progressive disturbances that occurred in the developing fetal or infant brain. The motor disorders of cerebral palsy are often accompanied by disturbances of sensation, perception, cognition, communication, and behaviour, by epilepsy and by musculoskeletal problems' *[2]. Children with CP are representative of many disabled children as they have a range of physical, intellectual, hearing, vision and communication impairments, with a wide range of severity. The severity of the motor impairment, and the associated cognitive communicative and behavioural impairments, are different for each child with CP [3]. Because the level of severity differs, their level of participation in everyday activities will vary greatly among children living with CP [4]. Even though the incidence of CP has not changed in the past 20 years its impact on quality of life of diagnosed children is not well understood.

The centre was one of a number of other centres who took part in the large study Sparcle [5]. Analysis on the participation of the total Sparcle population found frequency of participation varied between countries; children with CP participated less frequently in some areas, but not all, compared to the general population [6]. This paper outlines the results from 2 instruments from that study: The Frequency of participation questionnaire (FPQ) measuring participation in everyday activities and the KIDSCREEN questionnaire measuring quality of life in children. Children with CP have a relatively stable impairment where participation and quality of life are influenced by social, educational and environmental factors, as well as by medical interventions. The age group 8–12 years was targeted because it was much less studied than other age groups of children.

Measuring participation and its frequency is important as it adds an important dimension to whether or not a child accomplishes participation. The FPQ was designed to examine participation i.e. the actual accomplishment of the participation in a meaningful way. The instrument allows comparison with the general population and allows examination of participation independent of assistance. Domains in the International classification of functioning, disability and health (ICF) [7] for activity and participation are one in the same. The ICF introduced the concept of participation, defining it as involvement in life situations [7-9] leading to a greater sense of identification and belonging [10]. In the area of disability attention has recently focussed on the real life issues for a child living with a disability – *'disabled children have the same aspirations as all children; security, respect, opportunities to learn new skills, meaningful occupation and the possibility of contributing to the lives of others' *[11]. Clinicians therefore have to work towards real-life goals which include giving young children choices, and developing them as individuals. One way they can do this on a par is with adapted environments. One factor that influences participation is the barriers in the built environment. Adapted environments are essential for equal participation across all abilities. People with disabilities face barriers to physical activity in the social and built environments. To overcome these barriers people with disabilities need additional expenditure of resources to do so. Such were the findings of Kirchner et al [12] where problems with sidewalk pavement and poor drainage were the most cited environmental barriers. Participation is a valuable outcome measure for evaluating children's progress and for health services planning. Taking part in everyday activities for children with disabilities, on a par with children without disability, is vital for a sense of belonging within the community and an adapted environment facilitates this.

We know that children with physical and neurological disabilities enjoy the same activities as those without disabilities [13]. Children who are actively involved in school life can take advantage of educational and social benefits that arise from such an involvement. Children with CP are at increased risk of limitations to participation in every day activities [14,15]. Voorman et al [3] showed that activities and participation can be explained by the gross motor function level of a child, aged 9–16 years. Participation in everyday activities for children with disabilities is a goal shared by parents, service providers and organizations involved in children's rehabilitation [14].

Quality of life is used to describe a patient's condition at a level other than diagnosis [16,17]. The term quality of life has been defined as a *'subject-centred or individually appraised perspective on health *[18].*' *Work with children with CP is only now coming to the forefront [18]. Quality of life is usually described as an overall assessment of well-being across various domains [19]. Examples of domains that are covered when exploring quality of life are physical well-being, social well-being, emotional well-being, school, access to services, and acceptance by others [19]. Bjornson and Mc Laughlin [20] found fewer than 5% of the 1365 published measures of Quality of Life were applicable to children. The KIDSCREEN instrument has changed this by using the psychometrically valid and rigorously tested instrument on children [21,22]. Quality of life has been captured by this new European questionnaire – KIDSCREEN developed by taking account of the views of children and emphasising perception of psychosocial aspects of well-being rather than functioning or symptoms [21,6].

The aim of the study was to examine the frequency of activities by children in mainstream schools with and without CP. We also studied quality of life in children with CP, as reported by their parents or foster parents, and the relation between participation in everyday activities and their quality of life. In particular the relationship between participation in everyday activities and quality of life has not been addressed in previous studies. Specific research questions were – Does severity of impairment associated with CP impact on participation in everyday activities? Among children with CP, does participation in everyday activities affect quality of life after adjusting for severity of impairment?

## Methods

This was a cross-sectional study of children with and without CP based on questionnaires completed by parents/foster parents. Ethical approval was received from the National Research and Ethics Committee of the service provider. All parents gave written informed consent for the study subjects at the time of first contact.

### Participants

#### Children living with CP

The South of Ireland Cerebral Palsy Register (SICPR) is a register of all child residents of the counties of Cork and Kerry who, at the age of four years or older, were diagnosed as living with CP. During the study period (2004–2005), the families of all 120 children on the SICPR aged 8–12 years were contacted, first by telephone and then by letter. Ninety-eight parents participated (response rate = 82%) and completed structured questionnaires that measured participation, quality of life and impairment and a range of other factors, in the presence of the study researcher, usually in their home and taking a duration of 90–120 minutes.

#### Children not living with CP

Via the school principals, the Frequency of Participation Questionnaire (FPQ) was sent to the parents of all 650 children aged 8–12 years attending two Cork schools. A total of 448 completed questionnaires were received by the researcher (response rate = 69%). None of the children had a physical disability.

### Measures

#### Participation in everyday activities

The ICF recognises potential overlap in the concepts of activity with participation and hence classifies it across the same domain. The Sparcle group considered the concepts to be separate and wanted to examine participation separately independent of adaptations or assistance required by a child in doing the activity.

Frequency of participation (FPQ) in 14 everyday activities was measured using the FPQ instrument, an instrument developed by Sparcle from the Life Habits questionnaire [23,24]. The Life-H instrument was used as the conceptual framework for the frequency items. It was designed for disabled children and has been used in children with CP [25]. Our instrument differs from the Life-H because it captures how frequently activities are done. The Sparcle group chose the items from the Life-H instrument so that they would be relevant to the general population also. The FPQ has face validity as the questions were derived from the content of the Life-H instrument. We did not undertake intra and inter observer reliability. The 14 FPQ activities are: eating out, leisure, computer use, helping with housework, riding a bicycle, tricycle or wheelchair, running errands, joining organized activities outside school (community), school activities, playing sports, playing non-sports, watching sports, doing arts and crafts, going to cinema (culture), and taking part in tourist activities. Frequency of participation in each activity was assessed using a six-point Likert scale (scored 0–5): never, less than once a month, about once a month, once every two weeks, about once a week a few times a week. Because of limited numbers at some levels of participation, each of the 14 items was collapsed to two levels for the data analysis relating to the 14 activities. The sum of the original 14 items (scored 0–5) was used to give an overall measure of participation in everyday activities (possible range 0–70). For respondents who failed to answer one, two or three items, the average of the answered items was imputed for the unanswered items. Cronbach's alpha was 0.63 for the overall measure of participation which indicates a moderate level of reliability [26,27]. Overall participation scores followed a Normal distribution.

#### Quality of life

Quality of life was measured using KIDSCREEN, a 52-item generic health-related quality of life measure applicable to healthy and chronically ill children and adolescents aged 8–18 years and designed for child or parent report [21]. The KIDSCREEN instrument was used because it assesses across both healthy and ill children. It has been well validated psychometrically with 22,110 European children from the general population [21]. KIDSCREEN assesses ten domains of quality of life: physical well-being, psychological well-being, moods and emotions, self-perception, autonomy, parental relations, financial resources, social support and peers, school environment and social acceptance (bullying). The 52 items have a five-point Likert scale with two sets of possible responses: never, seldom, quite often, very often, always and not at all, slightly, moderately, very, extremely. For each domain, the relevant items are summed and scaled to yield a score in the range 0–100 with higher scores indicating better quality of life. Cronbach's alpha was greater than 0.7 for all ten quality of life domains except one at 0.69. These results suggest a high level of reliability for the domains [26,27]. Domain scores did not follow a Normal distribution because of the ceiling effect whereby high proportions of the children scored close to 100.

#### Level of impairment of children living with CP

Parents provided information about their child's gross motor function [28] and fine motor function [29]. CP type was available from the SICPR. Seizure activity, vision level, hearing level, feeding, communication and intelligence quotient (IQ) were recorded. Gross motor function and two-hand fine motor function were recorded according to the Gross Motor Function Classification System (GMFCS) and the Bimanual Fine Motor Function (BFMF) level. For the latter, levels I and II and levels III and IV were collapsed.

An overall, three-category measure of impairment was derived from the core impairment variables of gross motor function and IQ. Mild impairment was defined as GMFCS level I-III and IQ > 70. Moderate impairment was indicated by GMFCS level IV-V or IQ <= 70. Severe impairment was defined as GMFCS level IV-V and IQ <= 70.

### Statistical analysis

Data were analysed using SPSS v14. For the total study sample (i.e. children with CP and non-CP children), chi-square tests were used to assess the association between level of impairment and level of participation in each of the 14 everyday activities. Because overall participation scores followed a Normal distribution, the parametric t-test and one-way analysis of variance (ANOVA) were used to assess between-group differences with respect to two groups and more than two groups, respectively. Following statistically significant one-way ANOVA tests, Tukey's post hoc tests were used to identify the differing pairs of groups. A multiple linear regression model was estimated with overall participation in everyday activities as the dependent variable and gender, age and level of impairment as the independent variables. Diagnostic tests were used to check for violations of the assumptions inherent in linear regression models.

For the sample of children with CP, Mann-Whitney and Kruskall-Wallis tests were used to examine between-group differences in relation to the ten quality of life domains between two groups and more than two groups, respectively. For each quality of life domain, a multivariate linear regression model was estimated with quality of life as the dependant variable and gender, age, level of impairment and overall participation as the independent variables. Diagnostic tests were used to check for violations of the assumptions inherent in linear regression models.

## Results

The gender balance in the samples of children with and without CP were similar, slightly more boys than girls. The age distributions showed some variation (Chi-square = 9.717, df = 3, p = 0.021) with children aged 8–9 years making up 41% of the CP sample and 29% of the Non-CP sample. Half were diagnosed spastic unilateral, 37.8% (37) bilateral, with 6.1% (6) in both dyskinetic and ataxic CP (Table 1).

**Table 1:** Characteristics of the non-cerebral palsy (Non-CP) and cerebral palsy (CP) children

**Characteristics**	**Non-CP** **N (%)**	**CP** **N (%)**
**Gender**		
Male	208 (46.4%)	53 (54.1%)
Female	240 (53.6%)	52 (53.1%)
Age		
8–9 years	128 (28.6%)	40 (40.8%)
10 years	109 (24.3%)	20 (20.4%)
11 years	123 (27.5%)	15 (15.3%)
12 years +	88 (19.6%)	23 (23.5%)
**Classification (Gross Motor Function) levels I–V**		
I		37(37.8%)
II		22 (22.4%)
III		11 (11.2%)
IV		12 (12.2%)
V		16 (16.3%)
**CP Type**		
Spastic unilateral		49 (50.0%)
Spastic bilateral		37 (37.8%)
Dyskinetic		6 (6.1%)
Ataxic		6 (6.1%)
**Bimanual Fine Motor Function**		
Without Limitation		45 (45.9%)
Both Hands limited in fine skills or child needs help with tasks		33 (33.7%)
Child needs help and adapted equipment or total human assistance		20 (20.4%)
**Seizures**		
No Seizures (either with or without medication)		82 (83.6%)
Seizures		16 (16.4%)
**Feeding**		
No problems		73 (74.5%)
Feeds orally with difficulty, or by tube		25 (25.5%)
**Communication**		
Normal		66 (67.3%)
Difficulty but uses less speech		8 (8.2%)
Uses non-speech for formal communication		13 (13.3%)
No formal communication		11 (11.2%)
**Intellectual impairment**		
None or mild (IQ >70)		54 (55.1%)
Moderate or severe (IQ <= 70)		43 (43.9%)
Missing		1(1%)
**Hearing**		
Does not need hearing aid		96 (98%)
Needs hearing aids due to profound or severe loss >70 decibels		2 (2%)
**Vision**		
Has useful vision		96 (91.8%)
Blind or no useful vision		8 (8.2%)

### Participation in everyday activities

Level of impairment was significantly associated with participation in 11 of the 14 everyday activities examined (Table 2). No evidence of association was found in relation to eating out, riding a bicycle/tricycle/wheelchair and engaging in school activities. Using a computer was more common among the children with CP irrespective of their level of impairment. However, in general, increasing impairment was associated with decreasing participation.

**Table 2:** The association between participation in everyday activities and level of impairment

		**Non-CP**	**Mild disability**	**Moderate disability**	**Severe disability**	p-value*
**Eat out**	No more than once per month	242 (54.0%)	26 (50.0%)	10 (43.5%)	16 (69.6%)	0.308
	More than once a month	206 (46.0%)	26 (50.0%)	13 (56.5%)	7 (30.4%)	
**Leisure**	< few times per week	10 (2.2%)	3 (5.8%)	3 (13.0%)	4 (17.4%)	**<0.001**
	Few times per week	438 (97.8%)	49 (94.2%)	20 (87.0%)	19 (82.6%)	
**Computer**	< few times per week	254 (57.2%)	18 (34.6%)	6 (26.1%)	9 (39.1%)	**<0.001**
	Few times per week	190 (42.8%)	34 (65.4%)	17 (73.9%)	14 (60.9%)	
**Housework**	< few times per week	217 (48.7%)	25 (48.1%)	18 (78.3%)	19 (82.6%)	**<0.001**
	Few times per week	229 (51.3%)	27 (51.9%)	5 (21.7%)	4 (17.4%)	
**Riding a bike**	< few times per week	279 (62.8%)	28 (53.8%)	13 (56.5%)	9 (39.1%)	0.089
	Few times per week	165 (37.2%)	24 (46.2%)	10 (43.5%)	14 (60.9%)	
**Run errands**	< once per week	192 (43.5%)	26 (50.0%)	16 (69.6%)	16 (69.6%)	**0.009**
	At least once per week	249 (56.5%)	26 (50.0%)	7 (30.4%)	7 (30.4%)	
**Community**	< few times per week	225 (51.3%)	35 (67.3%)	21 (95.5%)	23 (100%)	**<0.001**
	Few times per week	214 (48.7%)	17 (32.7%)	1 (4.5%)	0 (0%)	
**School activities**	< once per month	275 (63.7%)	36 (73.5%)	15 (68.2%)	17 (77.3%)	0.330
	At least once per month	157 (39.3%)	13 (26.5%)	7 (31.8%)	5 (22.7%)	
**Play sports**	< few times per week	91 (20.7%)	24 (47.1%)	18 (81.8%)	19 (82.6%)	**<0.001**
	Few times per week	349 (79.3%)	27 (52.9%)	4 (18.2%)	4 (17.4%)	
**Play non-sports**	< few times per week	193 (43.8%)	19 (36.5%)	12 (54.5%)	17 (77.3%)	**0.008**
	Few times per week	248 (56.2%)	33 (63.5%)	10 (45.5%)	5 (22.7%)	
**Watch sports**	< few times per week	230 (52.2%)	38 (74.5%)	21 (91.3%)	21 (91.3%)	**<0.001**
	Few times per week	211 (47.8%)	13 (25.5%)	2 (8.7%)	2 (8.7%)	
**Do crafts**	< few times per week	233 (52.6%)	37(72.5%)	19 (82.6%)	17 (73.9%)	**<0.001**
	Few times per week	210 (47.4%)	14 (27.5%)	4 (17.4%)	6 (26.1%)	
**Culture**	< Once per month	127 (28.5%)	18 (35.3%)	13 (56.5%)	18 (78.3%)	**<0.001**
	At least once per month	318 (71.5%)	33 (64.7%)	10 (43.5%)	5 (21.7%)	
**Tourist**	< Once per week	254 (57.1%)	39 (75.0%)	19 (86.4%)	15 (68.2%)	**0.004**
	At least once per week	191 (42.9%)	13 (25.0%)	3 (13.6%)	7 (31.8%)	

* p-values based on chi-square tests with three degrees of freedom

Scores on the measure of overall participation in everyday activities ranged from 7 to 65 with a mean of 46.7 and standard deviation of 8.6. On average, girls had a marginally higher score than boys (mean: 47.3 v 46.2) but this difference was not statistically significant (t = -1.5, df = 541, p = 0.11). One-way ANOVA indicated that overall participation did not differ by age (F = 0.724, df = 3, 539, p = 0.538) but varied significantly by level of impairment (F = 68.445, df = 3, 539, p < 0.001). The post hoc tests indicated that the children at each of the four levels of impairment had significantly different levels of participation in everyday activities. Severely impaired children had the lowest level of participation (mean = 30.3), followed by those with moderate disability (mean = 35.4), those with mild impairment (mean = 43.9) and finally the non-CP children (mean = 48.5).

The multiple linear regression model with overall participation as the dependant variable and age, gender and impairment as the independent variables was statistically significant (F = 69.311, df = 3, 539, p < 0.001). More than a quarter of the variation in overall participation was explained by the model (Adjusted R^2 ^= 27.4%). The model indicated that overall participation was 1.2 higher in girls than in boys, a gender effect that just reached statistical significance (95% CI = 0.0 to 2.5; t = 1.970, p < 0.05). Participation was unrelated to age (t = 0.640, p = 0.523). Independent of age and gender, there was highly significant evidence of a graded effect of impairment on participation (t = 14.277 p < 0.001). From one level of impairment to the next (i.e. from non-CP to mild to moderate to severe CP), there was a 6.0 decrease (95% CI = -6.9 to -5.2) in a child's overall level of participation in everyday activities (Table 3).

**Table 3:** Results of multiple linear regressions of quality of life domains on overall participation in everyday activities, gender, age and level of impairment

**Quality of life domain**	**F**	**df**	**p-value**	**R^2 ^(%)**	B*	95% CI*	t*	p-value*
Physical well-being	3.69	4, 85	0.008	14.8	7.8	1.3 – 14.3	2.4	0.019
Psychological well-being	1.51	4, 89	0.206	6.4	4.4	-0.5 – 9.4	2.5	0.079
Moods & emotions	1.59	4, 86	0.183	6.9	4.5	0.4 – 8.6	2.2	0.031
Self perception	0.45	4, 84	0.769	2.1	0.8	-4.1 – 5.7	.33	0.741
Autonomy	0.86	4, 84	0.492	3.9	-0.2	-6.4 – 6.0	-.06	0.952
Parent relation & home life	0.94	4, 88	0.446	4.1	3.7	-1.5 – 9.0	1.4	0.158
Financial resources	0.61	4, 59	0.656	4.0	5.3	-6.2 – 16.7	.131	0.361
Social support & peers	8.51	4, 84	<0.001	28.9	13.9	7.7 – 20.0	4.4	<0.001
School environment	2.73	4, 86	0.034	11.3	3.7	-1.1 – 8.6	1.5	0.129
Social acceptance & bullying	0.81	4, 87	0.524	3.6	-0.3	-5.1 – 4.5	-.12	0.903

* Unstandardised coefficient and association statistics for overall participation after adjustment for gender, age and level of impairment

### Quality of life in children with CP

**Figure 1:**
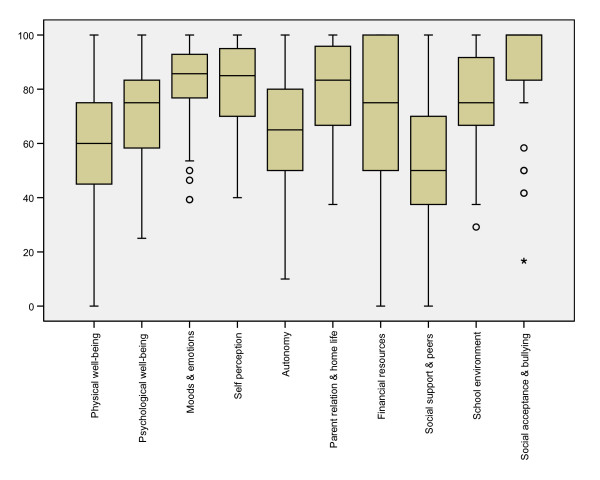
Box-plots of the 10 quality of life domains in 98 children with CP aged 8–12 years.

The distribution of scores on the ten domains of quality of life is illustrated in Figure 1. Ceiling effects, in that a high proportion of the children scored at or close to the maximum value, were evident in relation to Social acceptance and bullying, Financial resources, Self perception, Parent relation and home life, Social environment and Mood and emotions.

Increased severity of impairment was associated with significantly diminished quality of life but only in relation to two domains – Physical well-being (p < 0.05) and Social support and peers (p < 0.01). Respectively, children with mild, moderate and severe impairment had median scores of 65.0, 57.5 and 45.0 for Physical well-being and 62.5, 50.0 and 40.0 for Social support and peers.

Independent of gender, age and level of impairment, overall participation in everyday activities had a significant effect on three quality of life domains. A one-unit increase in participation was associated with increases of 7.8, 4.5 and 13.9 in quality of life related to Physical well-being, Moods and emotions and Social support and peers, respectively.

## Discussion

### Key findings

There was a stepped decrease in participation and quality of life from that of children without CP to those with mild, moderate and severe CP. Of the 14 everyday activities examined in the study, nine were affected by how impaired the child with CP was. The more impaired the child was the more affected they were in everyday activities. Girls had a slightly higher level of participation than boys in both samples, CP and Non-CP. There was a graded change in the level of impairment with the mean 43.89 for the mildly affected and 30.32 for those severely affected. In essence the mildly affected group was significantly more active than groups moderately impaired or the severely impaired group, the moderately impaired group was not significantly different than the severely impaired group. In other words the magnitude of the difference between the groups is quite small; approximately 10% difference between the mean score of those mildly affected and those living without a disability.

### Limitations

It is a weakness of the study that we did not include social class or income as a covariate in relation to the CP children. Income has been examined previously [14] and should be routinely included in Registry-based studies as income information is generally available on registers worldwide. When the non-CP children were examined they were found to be of average social class level and there was no evidence of either deprivation or high social class on visitations.

The sample was taken from a defined geographical area where the same services were available to the families of the CP children. Carrying out such a study on a national basis would have resulted in a more representative sample in the context of varied services.

The focus on measuring frequency of participation alone in the non CP children is a limitation of the study. In hind sight quality of life could have been measured and explored in both samples to enable a direct comparison across both populations of CP and non CP children in the age group.

The KIDSCREEN domains were not normally distributed which raises the question of suitability of using linear regression. Diagnostic tests were carried out following the linear regression analyses, the results of which showed no violation of the assumptions related to the model.

Despite these limitations the results contribute towards an understanding of the levels of participation and quality of life of children living with CP in Ireland.

This study makes an important and novel contribution to the literature and has critical implications for policy and practice in Ireland. It adds a further important dimension to whether Irish children participate and to what extent.

Beckung [28], Shenker [8] and Voorman [3] all found that impaired motor abilities explained a significant amount of the level of participation among children living with CP; our evidence showed the same; that as one moves from one level of impairment to another the average participation score is reduced by -6.97. In essence moving from one level of disability to the next a score of -6.97 comes off ones FPQ score. One area that can help with a child's level of impairment is adaptations in the environment. If the local health service provider provided for adaptations with the child's environment it would improve access and participation.

When we look at the two populations (CP and Non-CP) participation rates do not compare; the mean participation score moves gradually from 45–46–47 improving with age in the Non-CP population but in the CP population this is not the case, rather it moves 38–41–35–38. Anecdotally it is surprising to find that it was only 3 domains that were significant. And yet if we compare the Irish children with CP to the Swedish and Italian children with CP, Irish children participated less in most domains [6]. It was only in the Danish dataset that both CP and non CP children were on a par. Denmark is considered the front runner for valuing inclusion in disabled children [30]. All areas in Denmark have equal access [31]. One of the structures in place to facilitate this is public transport. Ireland must prioritise access for all.

The analysis of the full Sparcle dataset [6] found that in nearly all areas children with CP participated less than those not affected. But when in school all levels of impairment participated as much or more when activities were organised by the school [6]. This shows again that when the adaptations and systems are in place the impact of the impairment is lessened.

The research achieved what it set out to achieve, specifically to explore the severity of impairment of children diagnosed with CP, how it impacts on their quality of life, how the severity of their impairment impacts on their frequency of participation in activities and lastly to examine if participation in activities differed between children with CP and children in mainstream schools. It can be seen from the work that only in the severely affected children is both participation and quality of life affected. Therefore the hypothesis has to be accepted in that impairment does impact on quality of life and participation levels and that participation does differ between children living with CP and those age matched children without CP, if only marginally.

### Quality of life

The parents reported that the children with CP generally had a high quality of life. Increased impairment was associated with diminished quality of life in just two domains; Physical well-being and Social support and peers. However, independent of gender, age and level of impairment, overall participation in everyday activities significantly increased quality of life in these two domains and in the domain Moods and emotions. The low Physical well-being and Social support and peers domains means that the parents of these children think the children are lonely and find it difficult to make friends and communicate with their peers. This difficulty could be attributable to the environmental adaptations necessary to make friends such as accessing cinemas and restaurants to hang out with friends. This research will not change attitudes towards young people but could facilitate access to an improved social life by raising the awareness that they are lonely and do have physical impairments that stop them from getting places. Adaptations could improve their quality of life even further.

Although several studies have reported quality of life results for children with CP [20,30] few studies have assessed parent reported quality of life across a comprehensive set of subject domains for a group of children that are representative in all levels of severity in CP affected children. Another implication of our findings is that we must not consider parent reports equivalent to child's' report. It simply reflects a different perspective [6]. In the Sparcle paper most of the children were too severely affected to self report emphasising the importance of some report on the situation being essential.

## Conclusion

To our knowledge, this study is the first of its kind in Ireland looking at participation rates and quality of life in a population of children living with CP. It is the first positive news parents of Irish children with CP have received to date. When diagnosed it is now possible to tell parents of children with CP that in 9 activities children with CP participate well in everyday activities. Compared with the mainstream non affected population, children with mild CP compare well and do participate admirably with their peers. The findings concurred with similar work across all countries within the Sparcle project [6]. This is new knowledge for Irish parents and policy makers in the Health Care Service. It adds to the ongoing process of trying to better understand CP and its impact on the lives of those affected by it. The outcomes of this research recommend that parents, clinicians and educators of children with CP continue to encourage children with disabilities to take part in daily activities along side their mainstream peers in school and in their community. This can be done with support from local health services in funding adaptations. These results allow for informed service planning in the near future. The adaptations will encourage families to stay in the mainstream school system leading ultimately to a better quality of life for all concerned.

Further studies, based on longitudinal designs, should be carried out to determine the quality of life, participation levels and to identify the factors that predict the course of functioning in adolescents and adults with CP in the years ahead. These studies need to be done both nationally and internationally for the development of intervention programmes, policy and for planning the services that are needed for children, adolescents and adults and their families living with CP.

## Competing interests

The authors declare that they have no competing interests.

## Authors' contributions

VMM and PC hereby certify that they had access to all the data from the study and take responsibility for the integrity of the data and the accuracy of the analysis. IJP supervised the work. All authors (VMM, PC, IJP) contributed to the design of the study, interpretation of the data and read the manuscript critically. VMM is responsible for the writing. All authors' (VMM, PC and IJP) have read and approved the final script.

## Pre-publication history

The pre-publication history for this paper can be accessed here:


